# *Tn*P Peptide Suppresses Experimental Autoimmune Encephalomyelitis (EAE) in a Preclinical Mouse Model

**DOI:** 10.3389/fimmu.2022.857692

**Published:** 2022-03-24

**Authors:** Carla Lima, Adolfo Luis Almeida Maleski, Jefferson Thiago Gonçalves Bernardo, Vitor Cataldi Zelli, Evilin Naname Komegae, Monica Lopes-Ferreira

**Affiliations:** Immunoregulation Unit of the Laboratory of Applied Toxinology (CeTICs/FAPESP), Butantan Institute, São Paulo, Brazil

**Keywords:** EAE, synthetic peptide, fish venom, *Tn*P, pre-clinical, disease-modifying therapies

## Abstract

*Tn*P is a family of patented synthetic peptides which is in a preclinical development stage with valuable potential therapeutic indication for multiple sclerosis (MS), an autoimmune demyelinating disease of the central nervous system (CNS). The use of a preclinical animal model, such as experimental autoimmune encephalomyelitis (EAE) has deepened our knowledge of the immunomodulatory functions of *Tn*P as a drug. We have shown that *Tn*P possesses a disease suppressive function in EAE, ameliorating disease severity by 40% and suppressing the accumulation of T helper (Th)1- and Th17-producing lymphocytes (by 55% and 60%, respectively) in CNS along with activated microglia/macrophages populations (by 33% and 50%, respectively), and also conferred a protective effect anticipating the remyelination process to day 66 compared to day 83 of untreated cuprizone-mice. Here we expanded our knowledge about its effects compared with current first-line disease-modifying therapies (DMT). We demonstrated that prophylactic treatment with *Tn*P generated similar protection to betaseron (30%) or was more effective than glatiramer (44% versus 6%) or fingolimod (50% versus 19%) against the development of clinical symptoms. Although *Tn*P controlled the leukocyte infiltration (87% versus 82%) into demyelinated areas of the spinal cord in the same way as betaseron and fingolimod, it was more effective (72% to 78% decrease) in the long-term control of neuronal degeneration compared to them. Also, when compared to glatiramer, *Tn*P was more efficient in reversing leukocytes infiltration into the spinal cord (55% versus 24%), as well as induced a higher percentage of regulatory cells in spleen (2.9-fold versus 2.3-fold increase over vehicle-treated EAE mice) an in the spinal cord (8-fold versus 6-fold increase over vehicle-treated EAE mice). This specialized *Tn*P profile for inducing immune tolerance and neuronal regeneration has significant therapeutic potential for the treatment of MS and other autoimmune diseases.

## Introduction

Peptides as drugs have advanced and continue to grow with scientific innovation, expanding into new indications and molecular targets. Peptides represent a small portion (2%) of the world drug market, but together successful market peptides such as Copaxone (glatiramer acetate), Lupron, Zoladex, Sandostatin, and Velcade totaled about $25 billion in sales in 2018 (1).

There are currently ∼105 approved peptide drugs on the market, ∼200 in clinical development and ∼600 in the preclinical discovery stage. 86% of peptide drugs with therapeutic purposes or as approved molecular probes and diagnostic tools are of natural origin or synthetic derivatives from a natural source and the vast majority of them have a molar mass <2000 g/mol. The peptide market is growing twice as fast as the rest of the drug market, suggesting that peptides may soon occupy an ever larger niche in the therapeutic arsenal for metabolic, central nervous system (CNS), oncology, cardiovascular and chronic inflammatory diseases (2).

The oceans are an exceptionally rich source of bioactive metabolites, including the *Tn*P peptide extracted from the venom of the niquim, the Brazilian fish *Thalassophryne nattereri*. Our group identified and patented the family of analog peptides called the *Tn*P family which invention (3) relates to synthetic peptides containing a sequence of 13 L-amino acids and with a disulfide bond between Cys4 and Cys13 in its structure, and is in a preclinical development stage with valuable potential therapeutic indicated for chronic inflammatory diseases such as asthma and multiple sclerosis (MS). Measures aimed to improve the metabolic stability and half-life of the cyclic peptide *Tn*P were performed, including N-terminal amidation. Our data in the patent show that cyclic *Tn*P has remarkable resistance to the action of proteolytic enzymes such as trypsin and pepsin, and its high solubility allows it to be rapidly absorbed. In mice, we observed that immunization with *Tn*P at different doses, in the presence or absence of adjuvant, does not generate specific antibodies and, therefore, the molecule is not immunogenic.

The use of experimental systems, such as experimental autoimmune encephalomyelitis - EAE, has deepened our knowledge of the immunomodulatory functions of *Tn*P as a drug. In the extensive work carried out by our group (4), we found that subcutaneous treatment with *Tn*P successfully improves the severity of clinical signs of myelin oligodendrocyte glycoprotein (MOG)-induced EAE, delaying the onset of maximal symptoms (4 days) and decreasing the severity of symptoms by 40% compared with control EAE mice treated with vehicle alone. *Tn*P beneficially interferes with the immune circuit at various stages by partially IL-10-dependent mechanisms, including suppressing activation of conventional dendritic cells (DC) and providing the emergence of plasmacytoid DC and regulatory cells during the EAE induction phase; blocking the transit and infiltration of leukocytes into the CNS by suppressing matrix metalloproteinase (MMP)-9 activity and CD18 expression; blocking the reactivation and permanence of Th1 and Th17 lymphocytes in the CNS; the prevention of microglial expansion and macrophage infiltration into the CNS; favoring the localized increase in regulatory T cells; and finally, suppressing demyelination in the spinal cord of EAE mice and leading to accelerated remyelination in a cuprizone model.

In view of the advance in the preclinical development chain, the safety and biodistribution of *Tn*P were recently investigated using zebrafish as a model for preclinical toxicology studies. We reveal a broad therapeutic index with non-lethal doses ranging from 1 nM to 10 μM, without neurotoxicity or cardiotoxic effects. The low frequency of *Tn*P-induced abnormalities in embryos was associated with the high safety of the molecule and the ability of the developing embryo to process and eliminate it. *Tn*P crossed the blood-brain barrier without disturbing the development of the normal architecture of the forebrain, midbrain, and hindbrain (5).

Currently, the *Tn*P family invention is patented in Brazil (PI0602885-3A2, original #PI0703175-0, 09/04/2019); Europe – WO/2008/009085 (2008); United States – US20100144607 (2012); Canada - CA2657338 (2013); China - CN101511861 (2013); Hong Kong - HK1135406 (19/09/2013); India – IN94/MUMNP/2009 (2013); South Korea - KR1020090037900 (2014), and Japan – JP2010503613 (2014), and thanks to its low toxicity and pharmacological characteristics, it is presented as a candidate for the development of new pharmacological strategies to reduce the effects of multiple sclerosis. Here the aim of our work is to expand our knowledge about its effects compared with current disease-modifying therapies (DMTs).

## Results

### TnP Is More Effective Than Betaseron to Maintain Long-Term Neuronal Health

The first large scale human clinical trial in patients with relapsing-remitting MS (RRMS) using interferon beta (IFN-β) was published in 1993 and showed that relapse rates were reduced by 34% in high dose IFN-β1b and by 8% in lower dose compared to placebo group, and severity of relapses was also reduced (6).

**Figure 1:**
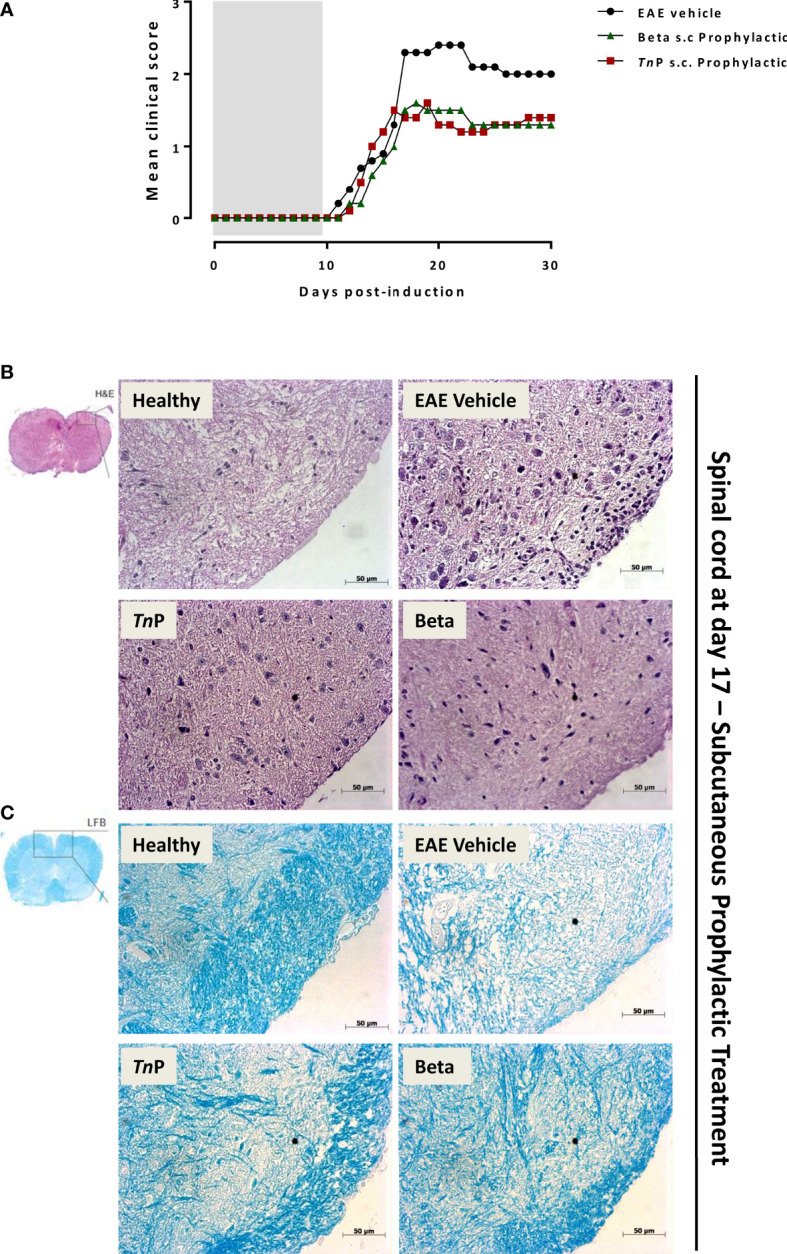
*Tn*P improves EAE clinical signs and controls inflammation and demyelination compared to betaseron. BL6 mice (n = 5/group) immunized with (MOG) peptide in incomplete Freund’s adjuvant added with *M*. *tuberculosis* were injected 2 times with *Pertussis* toxin after immunization. Mice were scored (0–5) daily for 30 d for evidence of clinical signs of disease **(A)**. Mice were s.c. treated with *Tn*P at 3 mg/Kg or betaseron at 10,000 UI/mice every other day starting at the day 0 of immunization until day 9 (prophylactic regimen). The EAE controls were injected with 0.9% saline alone (Vehicle). Spinal cords from healthy, vehicle-treated EAE, and EAE treated-mice were removed on the peak of disease (17) and stained in with H&E **(B)** in the upper panels or Luxol fast blue in the lower ones **(C)**. Representative sections are shown.

To study the manifestations of EAE as well as *Tn*P compared to betaseron subcutaneous (s.c.) prophylactic treatment in the CNS, we used the MOG_35–55_ peptide-induced EAE model in the susceptible strain (**Figures 1A–C**). Female C57BL/6J (BL6) mice induced with EAE and treated with vehicle exhibited chronic disease progression with onset around day 11 post-induction (p.i.), exhibiting mean maximal clinical score of 2.3 ± 0.09 at day 17 p.i. that was maintained in chronic phase, grade 2–2.1.

*Tn*P (3 mg/Kg) or betaseron (10,000 UI/mice) treatments started with s.c. daily application by 10 days during induction phase. EAE symptoms appeared on day 12 p.i. for both treated-groups (0.1 ± 0.01 and 0.2 ± 0.01, respectively). *Tn*P as well as betaseron both ameliorated the clinical manifestations of EAE decreasing by 30% the mean maximal clinical score from 2.3 ± 0.09 to 1.6 ± 0.02. *Tn*P delayed the onset of maximal symptoms by 2 days (19) and betaseron delayed the onset of symptoms by 1 day (18). The beneficial effect was stable over time and sustained until day 30 (**Figure 1A**).

Because immune cell infiltration of the CNS is considered a hallmark of EAE, we investigated the effect of *Tn*P compared to betaseron on leukocyte infiltration in the CNS by the analysis of hematoxylin and eosin (H&E, **Figure 1B**) or with Luxol fast blue (LFB, **Figure 1C**) paraffin sections at day 17. While vehicle-treated EAE mice presented extensive infiltration of inflammatory cells per mm^2^ (254 ± 4.3) and areas of demyelination (score 3), tissue from *Tn*P- (34 ± 2.3 and score 1.5) or betaseron-treated mice (45 ± 1.9 and score 1.2) lacked severe signs of inflammation with preserved myelinated areas.

**Figure 2:**
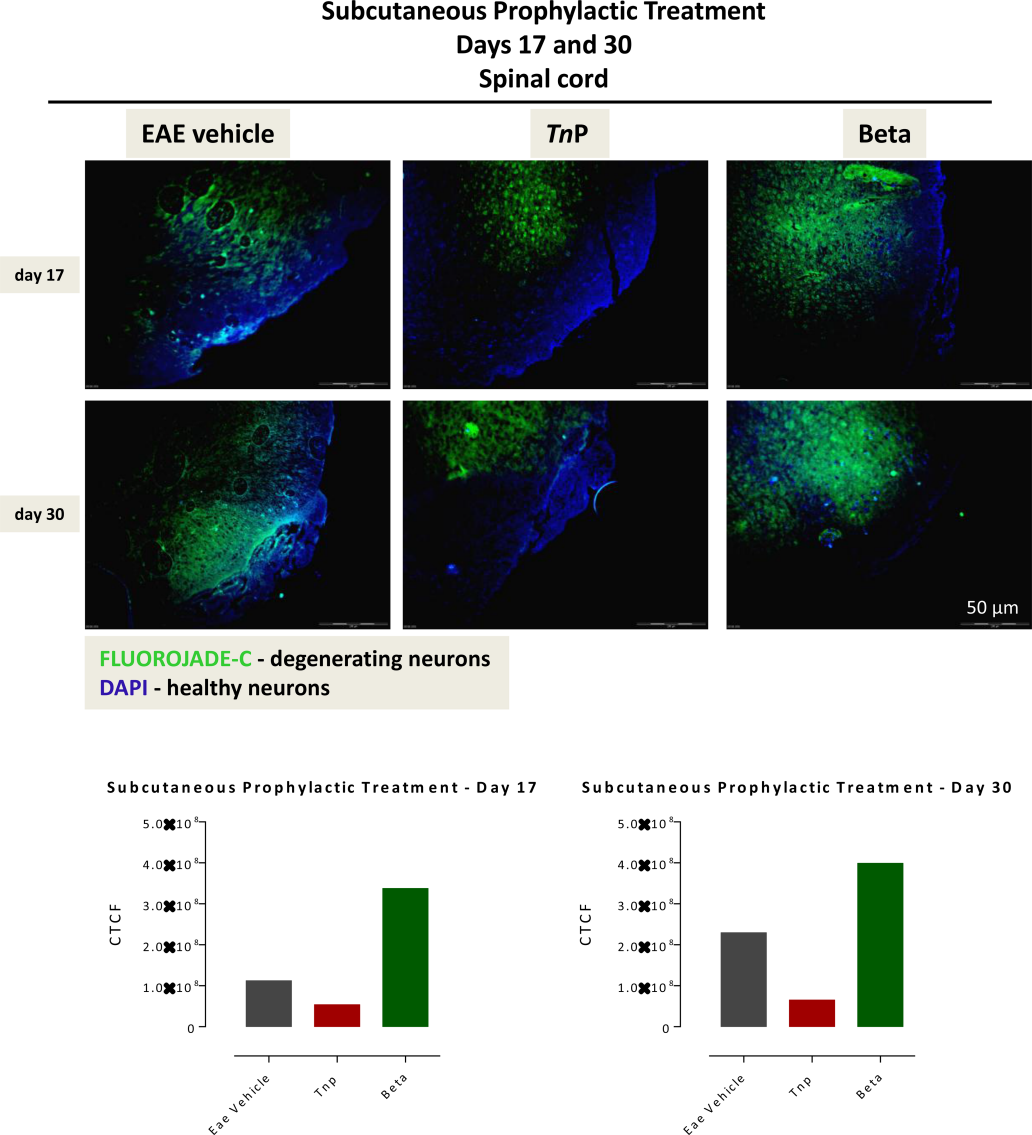
Subcutaneously *Tn*P treatment promotes the long-lasting survival of neurons in spinal cord. The 10 micron-thick slices of spinal cord of vehicle-treated EAE or *Tn*P- and betaseron-treated EAE mice removed at day 17 or 30 were prepared for immunofluorescent double-staining with Fluoro-Jade C (green) and DAPI (blue) for evaluation the degree of neurodegeneration in damage areas. Representative sections are shown. The fluorescence staining was measured in ImageJ Software v.1.8.0_172. The corrected total cell fluorescence (CTCF) was obtained utilizing the formula CTCF = Integrated Density – (Area of selected cell X Mean fluorescence of background readings).

IFNs do not directly exert a neuroprotective effect; however, through their direct effect on encephalitogenic CD4+Th1 cells, altering their activation state trigger a decrease in neuronal demyelination, which prevents further neuronal damage (7). Next, Fluoro-Jade C was used to evaluate the degree of neurodegeneration in damage areas of the spinal cord of vehicle- or treated-EAE mice and the corrected total cell fluorescence (CTCF) was obtained (**Figure 2**). Vehicle-treated EAE mice exhibited a significantly increased number of degenerating neurons on day 17 (1.12 ± 0.01). Degeneration progressively intensified up to day 30 p.i. in this group, from central to the peripheral areas (2.29 ± 0.01). The *Tn*P-treated group of mice showed a mild degeneration (0.54 ± 0.01) in the central region that remained similar until day 30 (0.65 ± 0.01). In contrast, the intense neuronal degeneration seen on day 17 (3.38 ± 0.01) in betaseron-treated group was observed to increase at day 30 (3.99 ± 0.01). In summary, these data show that subcutaneous treatment with *Tn*P was more effective in the long-term control of neuronal degeneration.

### The Capacity of Prevention of Microglial Expansion and Macrophage Infiltration, Expansion of Regulatory Cells Into the CNS Is Higher for TnP Than Glatiramer

Like IFN-β, glatiramer exerts broad immunomodulatory effects that are incompletely understood. In patients, glatiramer significantly reduced disease symptoms and development of new lesions by up to 30% in RRMS, although it showed no improvement in long-term efficacy on progression of disability (8). Next we examined the role of *Tn*P in the control of EAE symptoms compared with glatiramer in a prophylactic regimen of subcutaneous application.

**Figure 3:**
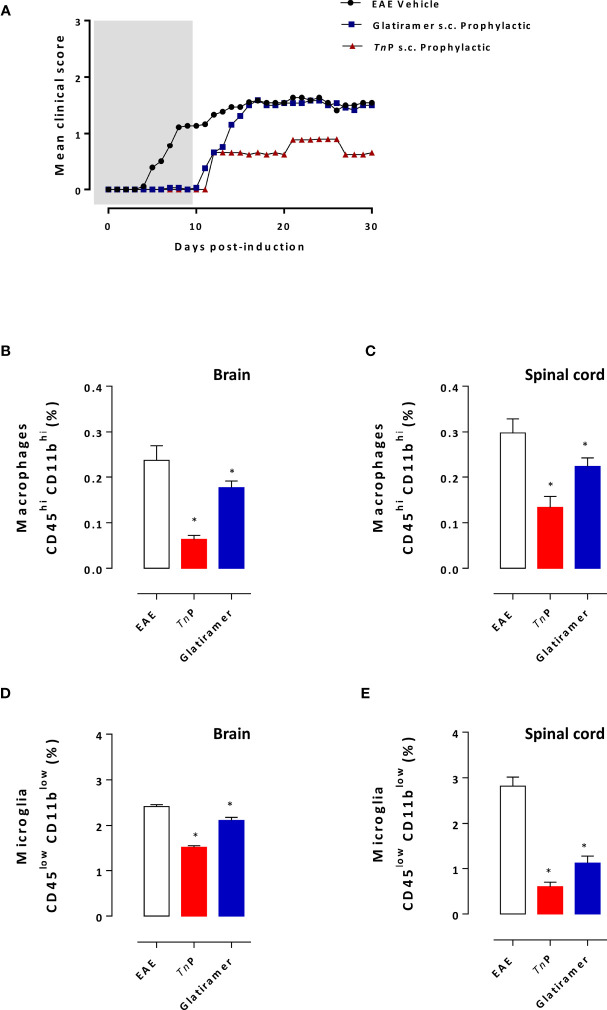
*Tn*P is more efficient than glatiramer in ameliorating the clinical signs of EAE, inflammation and demyelination. BL6 mice (n = 5/group) immunized with (MOG) peptide in incomplete Freund’s adjuvant added with *M*. *tuberculosis* were injected 2 times with *Pertussis* toxin after immunization. Mice were scored (0–5) daily for 30 d for evidence of clinical disease **(A)**. Mice were s.c. treated with *Tn*P at 3 mg/Kg or glatiramer at 2 mg/mice every other day starting at the day 0 of immunization until day 9 (prophylactic regimen). The EAE controls were injected with 0.9% saline alone (Vehicle). At day 17 post immunization, CNS-infiltrating leukocytes were isolated from pooled brain **(B**, **D)** and spinal cord **(C**, **E)** homogenates of all mice and the percentage of microglia (CD45lowCD11blow) and infiltrating macrophages (CD45^high^CD11bhigh) were analyzed by flow cytometry after acquisition of 100,000 events as shown in the schematic image of **Supplementary Figure 1A**. Values in the bar graphs are the mean ± SEM. *p < 0.05 compared with vehicle-treated EAE mice.

Mice that received *Tn*P showed substantially fewer neurological deficits by the measurement of the mean maximal clinical score of 0.9 ± 0.03 than mice that received glatiramer (1.5 ± 0.03) or vehicle-treated EAE mice (1.6 ± 0.01). The decrease in mean maximal clinical score induced by glatiramer was 6% in the first 5 days compared to 44% of decrease caused by *Tn*P treatment which lasted for 30 days. In addition, we observed that *Tn*P delayed the peak of symptoms by 4 days, while glatiramer accelerated it by 1 day (**Figure 3A**).

At the peak of disease (day 17), microglia and macrophage infiltrations were examined in the CNS of vehicle- or treated-groups. The percentage of CD45^high^CD11b^high^ macrophages infiltrating the brain or spinal cord decreased by 74% and 55%, respectively, after *Tn*P treatment compared to a 26% and 24% of decrease caused by glatiramer (**Figures 3B, C**). In addition, *Tn*P caused a 37% of decrease in CD45^low^CD11b^low^ microglia in the brain and 79% in the spinal cord compared to percentages of 12% and 60% induced by glatiramer (**Figures 3D, E**).

**Figure 4:**
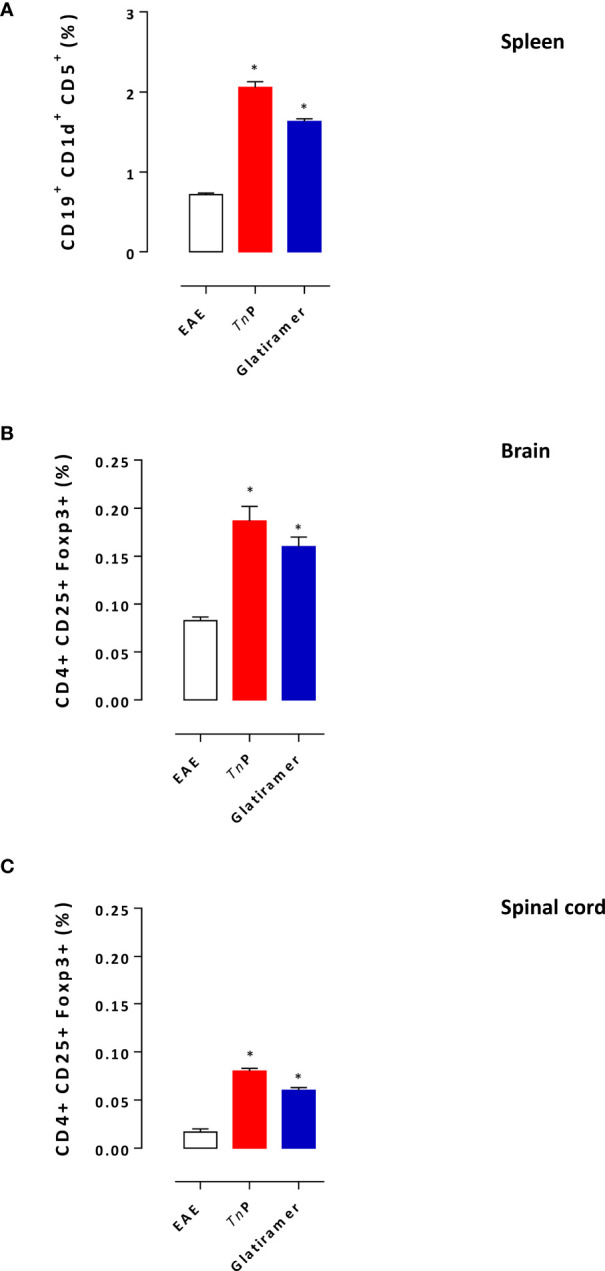
*Tn*P increases the expansion of regulatory cells. Splenocytes isolated at day 7 **(A)** and CNS-infiltrating leukocytes isolated at the peak of disease (17 day) from pooled brain **(B)** and spinal cord **(C)** homogenates of EAE mice treated with vehicle, *Tn*P or glatiramer were evaluated for the percentage of Breg or Treg after acquisition of 100,000 events as shown in the schematic image of **Supplementary Figure 1B, C**. Data represent mean ± SEM. *p < 0.05 compared with vehicle-treated EAE mice.

Glatiramer treatment results in the skewing of auto-reactive lymphocytes away from the pathogenic effector cell responses towards regulatory functions (9). The next step was to compare the ability of prophylactic treatment with *Tn*P to induce regulatory T and B cells in the presence of EAE. As showed in **Figure 4A**, an increase in the percentage of activated CD19^+^CD1d^+^CD5^+^B regulatory (Breg) during the priming phase was also observed in the spleen of *Tn*P- (2.9-fold) as well as glatiramer- (2.3-fold) compared to vehicle-treated EAE mice, consistent with a systemic effect of both treatments.

We also examined the percentage of conventional CD4^+^CD25^+^FOXP3^+^T regulatory (Treg) cells in the CNS (**Figures 4B, C**). A higher percentage of CD4+ Treg cells was detected in the CNS mainly in the spinal cord of *Tn*P-treated mice during the peak phase of the disease (2.3-fold and 8-fold increase), compared to a percentage (2-fold and 6-fold increase) of these cells in the CNS of glatiramer-treated mice.

### TnP Controls EAE Progression by the Maintenance of Long-Term Neuronal Health and Protects From Weight Loss

Fingolimod was granted Food and Drug Administration approval in 2010 for oral therapy (10). Fingolimod is effective in reducing clinical exacerbations and delays the disability progression in RRMS patients based on reduction of encephalitogenic lymphocytes egress from secondary lymphoid organs (11–14).

**Figure 5:**
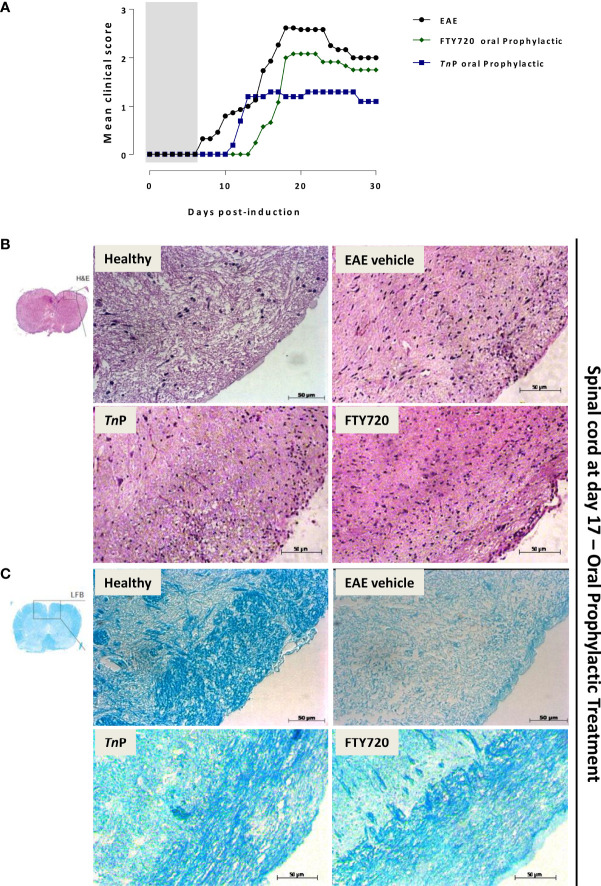
*Tn*P applied orally is more effective in the control of disease than fingolimod. BL6 mice (n = 5/group) immunized with (MOG) peptide in incomplete Freund’s adjuvant added with *M*. *tuberculosis* were injected 2 times with *Pertussis* toxin after immunization. Mice were scored (0–5) daily for 30 d for evidence of clinical disease **(A)**. Mice were administered by oral gavage with *Tn*P at 3 mg/Kg or fingolimod at 0.3 mg/Kg diluted in 0.9% saline every other day starting at the day 0 of immunization until day 9 (prophylactic regimen). The EAE controls were injected with 0.9% saline alone (Vehicle). Spinal cords from healthy, vehicle-treated EAE, and EAE treated mice were removed on the peak of disease (17) and stained in with H&E **(B)** in the upper panels or Luxol fast blue in the lower ones **(C)**. Representative sections are shown.

After onset at day 7, EAE progressed, and vehicle-treated mice reached the peak of the disease (mean = 2.6 ± 0.19) at day 18. After prophylactically administered oral *Tn*P, clinical signs became evident at 11 ± 1 p.i. and developed a disease that peaked at day 16 ± 1 p.i. with clinical score = 1.3 ± 0.09, indicating a decrease of 50%. In contrast, in prophylactically fingolimod-treated EAE mice, clinical symptoms were observed starting from 14 ± 1 p.i. with the maximal gravity (clinical score = 2.1 ± 0.03) being evident at day 19 p.i. ± 1, representing 19% of decrease. These data show that both treatments delayed onset of disease from 7 to 11 or 14, but only *Tn*P significantly reduced peak symptoms by 50% (**Figure 5A**).

In order to further analyze the impact of prophylactic treatment by *Tn*P or fingolimod on inflammation and myelin distribution, spinal cords from vehicle- and treated-mice were assessed by H&E (**Figure 5B**) and LFB (**Figure 5C**) staining. The cell infiltration was moderately prevented in the spinal cord white matter of *Tn*P (67 ± 3) or fingolimod-treated EAE mice (70 ± 4) when compared to the control or vehicle-treated EAE groups (159 ± 6). Seventeen days after EAE induction, demyelination was clearly evident in the white matter of spinal cord of vehicle-treated EAE mice. Levels of intense remyelination were observed after *Tn*P and fingolimod-treated mice.

**Figure 6:**
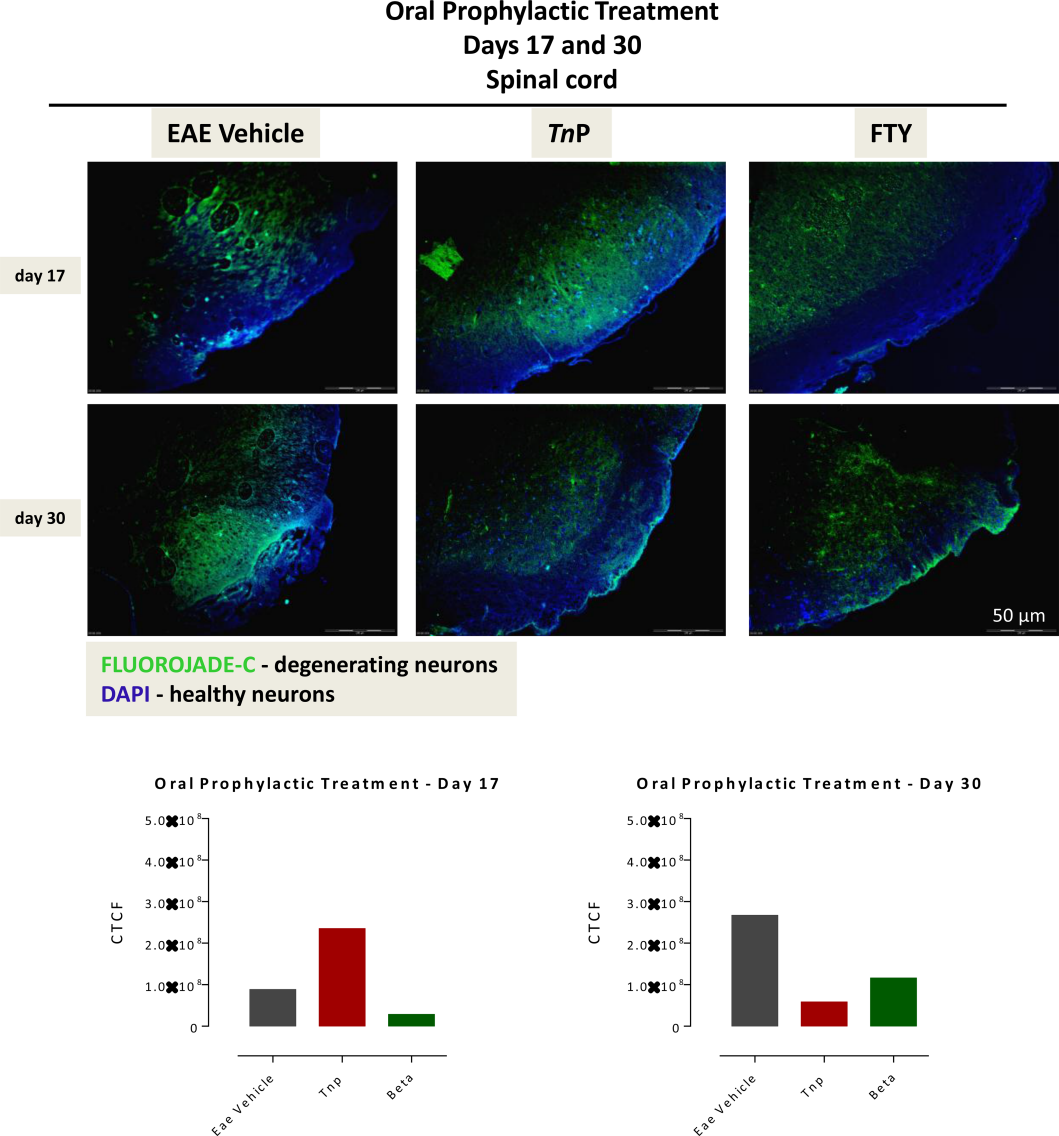
*Tn*P orally administered promotes the long-lasting survival of neurons in spinal cord. The 10 micron-thick slices of spinal cord of vehicle-treated EAE or *Tn*P- and fingolimod-treated EAE mice removed at day 17 or 30 were prepared for immunofluorescent double-staining with Fluoro-Jade C (green) and DAPI (blue) for evaluation the degree of neurodegeneration in damage areas. Representative sections are shown. The fluorescence staining was measured in ImageJ Software v.1.8.0_172. The corrected total cell fluorescence (CTCF) was obtained utilizing the formula CTCF = Integrated Density – (Area of selected cell X Mean fluorescence of background readings).

In addition, analysis of the spinal cord by Fluoro-Jade C revealed that the tissues of mice treated prophylactically with oral *Tn*P showed a large extent of degenerated neurons on day 17 (2.35 ± 0.01), compared to vehicle-treated EAE mice (0.88 ± 0.01) that were reduced dramatically until day 30 (*Tn*P: 0.58 ± 0.01 versus vehicle: 2.67 ± 0.01). Fingolimod-treated mice showed a smaller area of neuronal degeneration at day 17 (0.28 ± 0.01) compared to *Tn*P, but analysis of progression shows that the injury got worse after 30 days in fingolimod-treated mice (1.16 ± 0.01) (**Figure 6**). Although the number of degenerated neurons in the fingolimod-treated mice is lower than the vehicle-treated mice at day 30 by 56%, they are 2-fold higher than the *Tn*P-treated mice. Together, these data confirmed that *Tn*P promoted an improvement in long-term neuronal regeneration.

**Figure 7:**
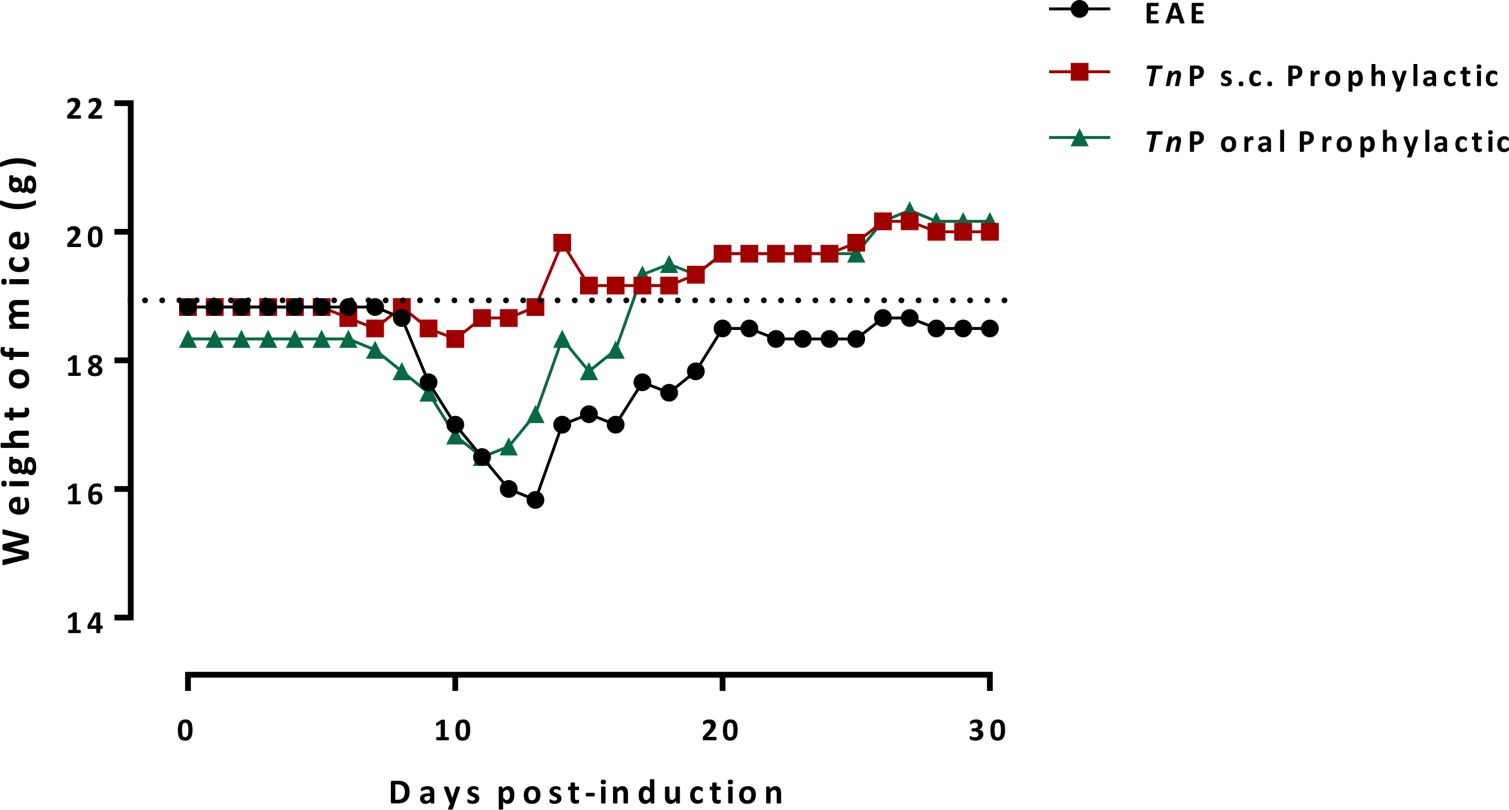
Subcutaneous or oral treatments with *Tn*P prevent weight loss at the effector phase and increase in the chronic phase of the disease. BL6 mice (n = 5/group) immunized with (MOG) peptide in incomplete Freund’s adjuvant added with *M*. *tuberculosis* were injected 2 times with *Pertussis* toxin after immunization. Mice were s.c. or orally treated with *Tn*P at 3 mg/Kg every other day starting at the day of immunization in the prophylactic regimen (day 0 to 9). The EAE controls were injected with 0.9% saline alone (Vehicle). Treated-mice weighed daily were compared to normal weight of vehicle-treated or healthy mice (dotted line).

Finally, all vehicle-treated EAE mice displayed a prominent body weight loss from day 10, onset of symptoms peaking on day 14. The low weight observed in EAE mice remained until day 21 when it slightly increased and stabilized. The improvement of clinical score induced by *Tn*P corresponded to an increase in body weight immediately after disease induction. The s.c. treatment of mice with *Tn*P was more effective than oral administration in improving weight loss as it prevented weight loss between days 10 to 21 and, unlike untreated mice, it induced weight gain from day 22 to 30. Oral *Tn*P-treatment of EAE mice induced partial protection between days 10 to 17, but from day 18, weight gain was similar to s.c. administration (**Figure 7**).

## Discussion

*Tn*P demonstrated high stability, high safety, low toxicity (5) and immunogenicity (3) added to the ability to cross the blood-brain barrier. These properties lead us to predict a potential ability of interaction with lipid membranes allowing cellular membrane crossing and access to intracellular targets (15, 16).

Here in this study, we extend our previous observations of the anti-inflammatory and neuroprotective effects of *Tn*P (4) as we provide consistent and stronger evidence by comparing it to commonly used drugs such as interferon beta-1 (IFNβ-1), glatiramer acetate, and the modulator of the sphingosine 1-phosphate receptor fingolimod (17).

Our results confirm that BL6 EAE mice treated prophylactically with subcutaneously or orally applied *Tn*P exhibited protection against the development of CNS symptoms and inflammation similar to betaseron or fingolimod, but *Tn*P was more effective than both in the long-term control of neuronal degeneration. Interestingly, we demonstrated that, when compared to glatiramer, *Tn*P was more efficient in reversing clinical symptoms, altering the fate of the leukocytes into the spinal cord, by either switching off pathogenic cells as macrophages and microglia, or by activating regulatory cells such as splenic Breg during the priming phase and Treg in the CNS in the chronic phase.

Recently, we conducted investigations to identify the molecular basis of the therapeutic effect of *Tn*P. Using a zebrafish model of neutrophilic inflammation, we found that *Tn*P treatment of inflamed-embryos was followed by up-regulation of only four known miRNAs and the mature dre-miR-26a-1 (miR-26a) was the most highly expressed. To confirm the involvement of miR-26a in the modulation of inflammation triggered by *Tn*P, we used a combination of gain- and loss-of-function approaches as mimetic RNA or morpholino (18).

The knockdown of miR-26a ubiquitously by morpholino resulted in a significant reduction of miR-26a in embryos, accompanied by impaired *Tn*P immunomodulatory function observed by the loss of control of neutrophil removal in response to inflammation; while overexpression by the use of mimetic mRNA increased the inhibition of neutrophilic inflammation promoted by *Tn*P. The remarkable importance of miR-26a was confirmed when the rescue strategy was used (18). With these results we identified miR-26a as an essential molecular regulator of the therapeutic action of *Tn*P.

Furthermore, exploring the possible multiple molecular gene-targets of miR-21, miR-122, miR-731, and miR-26 overexpressed by *Tn*P we concluded that, through this miRNA pathway, *Tn*P can regulate many genes in one pathway or even multiple cross-pathways, generating a tremendous impact on a complex regulatory network and, consequently, on the magnitude of neurogenesis and inflammation (19, 20).

These findings that demonstrate the multiplicity of action of *Tn*P are in line with the new trend towards peptide drug development for the immunotherapy of many diseases, such as MS (21, 22) which predicts that the ability of cyclic peptides to modulate the immune response is associated with their simultaneous action on multiple molecular targets (23).

In general, these DMTs target neuroinflammation, and are considered to have a marginal, if any, effect on neurodegeneration, which is the main underlying mechanism of disability progression in MS (9, 21, 24). Fingolimod was the first approved oral therapy for the relapsing form of MS. Experimental evidence increasingly supports a direct action of fingolimod on neural cells within the CNS, providing protection against the neurodegenerative component of MS (25).

However, what we demonstrate here is that mice treated with fingolimod had progressive worsening of neuronal degeneration in contrast to oral *Tn*P treatment. These findings showing the neuroprotective effect of *Tn*P suggest alternative and overlapping mechanisms, acting to block the interactions between auto-reactive leukocytes and vascular endothelial cells in the CNS thereby inhibiting transmigration of these cells; or by restoring normal axonal health and preventing neurodegeneration and accelerating the remyelination process.

In conclusion, the demonstrated ability of *Tn*P to modify the course of the disease and to improve symptoms, added to neuronal stability in preventing degeneration, highlights its applicability as a promising therapeutic management in the pre-symptomatic phase of MS preventing disease progression. *Tn*P appears to work at multiple stages in the course of the disease improving the robust pipeline of experimental oral therapies for MS.

## Materials and Methods

### Mice

Four to six-week-old female C57BL/6J (BL6) wild type mice were obtained from a colony at the Butantan Institute, São Paulo, Brazil. Mice were kept in the same SPF animal unit maintained in sterile micro-isolators with sterile rodent feed and acidified water and housed in positive-pressure air-conditioned units (25°C, 50% relative humidity) on a 12 h light/dark cycle. This study was carried out in strict accordance with the recommendations in the Guide for the Care and Use of Laboratory Animals of the Brazilian College of Animal Experimentation. The protocol was approved by the Committee on the Ethics of Animal Experiments of the Butantan Institute (#747/10; #1203/14; #1205/14).

### TnP and MOG Synthetic Peptides and Medicines

*Tn*P *trifluoroacetate compound* (P13821401, C_63_H_114_N_22_O_13_S_4_, purity 97.3%) was manufactured under the patent holder’s proprietary method (Laboratório Cristália Produtos Químicos Farmacêuticos LTDA). Myelin oligodendrocyte glycoprotein trifluoroacetate lyophilized powder (MOG)_35-55_ peptide (316131-3 part P1822131, MEVGWYRSPFSRVVHLYRNGK, 2581,4 Da e 95.2%) was purchased from GenScript (order 20615, Piscataway NJ USA). Copaxone (glatiramer acetate, P53888, P63924, or P63033) was purchased by Teva Pharmaceutical Industries Ltd (Petah Tikva, Israel). rhIFNβ-1b (betaseron) was purchased by Bayer Health Care Pharmaceuticals. Fingolimod (FTY720 hydrochloride, Gilenya; Novartis) was purchased by Cayman Chemical.

### Analysis of Clinical Signs of Active EAE

EAE was induced according to Mendel et al. (26). Briefly, four to six-week-old female BL6 (*n* = 5 per group) received a subcutaneous injection (s.c.) in the tail base of 300 µg of MOG_35–55_ per animal emulsified in 100 µL incomplete Freund’s adjuvant (IFC, 263910, Difco) containing 500 µg of *Mycobacterium tuberculosis* H37RA (231141, part 0239413, Difco) on day 0. Immediately thereafter and again 48 h later, mice received an intraperitoneal (i.p.) injection of 500 ng of *Pertussis* toxin (P7208, part SLBK1874V, Sigma-Aldrich, St Louis, MO, USA) diluted in 200 µL of sterile 0.9% saline. EAE progression was monitored for 30 d after immunization with MOG. Clinical sign scores of EAE were daily assigned as follows: 1, tail limpness; 2, impaired righting reflex; 3, hind limb paralysis; 4, hind- and forelimb paralysis; 5, death. The mean of monthly scores was calculated. Mice were weight every day. All behavioral measurements were done in awake, unrestrained, age matched female mice. All tests were performed in an appropriate quiet room between 10 am and 4 pm. If necessary, food was provided on the cage floor. Prior injection of *Pertussis toxin* mice were anaesthetized with isoflurane. A humane endpoint was fixed using specific parameters as follow: EAE mice consistently scored higher (≥4, complete hind limb paralysis or quadriparesis, and weight loss greater than 30%) were removed from the study and killed.

### Administration of TnP and Medicines

For subcutaneous treatment, mice (*n* = 5/group) were injected with 100 µL of *Tn*P at dose of 3 mg/Kg mice according to Komegae et al. (4); glatiramer at 2 mg/mice according to Aharoni et al. (27) or betaseron at 10,000 UI/mice according to Inoue et al. (28) at, all diluted in 0.9% saline on the back. For oral treatment, mice previously submitted to a 4-hour fast received 30 µL of *Tn*P at 3 mg/Kg or fingolimod at 0.3 mg/Kg mice according to Bonfiglio et al. (29), all diluted in 0.9% saline administered by oral gavage. Mice were treated every day from day 0 to 9 (Prophylactic treatment - during induction phase). The EAE controls were injected with 0.9% saline alone (Vehicle).

### Cell Preparation From Spleen and CNS

Healthy, vehicle-treated EAE, and EAE treated-mice (n = 5/group) were killed and spleens were removed at day 7 p.i. for analysis during the induction phase. The brain and the spinal cord were excised from mice perfused transcardially with ice-cold phosphate buffered saline (PBS) at peak of disease (17). Single-cell suspensions of splenic tissue were prepared by digestion with 1 mg/mL of type II collagenase (Roche) and 500 U DNase I (Sigma-Aldrich) following by mechanical disruption in GentleMacs dissociator (Miltenyi). Erythrocytes in spleens were lysed with 0.14 M NH4Cl and 17 mM Tris-Cl (pH 7.4). Additionally, the brain and spinal cord cell suspensions were prepared and centrifuged at 200 g for 10 min and resuspended in 4 mL of 30% isotonic Percoll (P1644, Sigma) diluted in HBSS and overlaid by equal volumes of 37% and 70% isotonic Percoll. The gradient was centrifuged at 800 g for 20 min and leukocytes were harvested from the 37% - 70% interface, washed, and counted.

### Flow Cytometry Analysis

Spleen, brain, and the spinal cord from healthy, vehicle-treated EAE, and EAE treated-mice were harvested and cell suspensions were prepared. Immune cell infiltration and resident microglia activation were assessed per cytofluorometric (FACS) by cytometer analysis. For surface staining, single-cell suspensions (1 x 106 cells in 100 µL) were treated with 3% mouse serum of naive mice and then incubated for 30 min in ice with specific anti-mouse antibodies (Abs) fluorochromes-conjugated or purified Abs followed by secondary Abs fluorochromes-conjugated purchased from BD Biosciences, R&D Systems or eBioscience: PerCP-Cy5.5-CD11b (550993, BD Biosciences), PE-CD11b (557397, BD), FITC-CD4 (553729, BD Biosciences), PE-CD4 (557308, BD Biosciences), PE-CD25 (553075, BD Biosciences), FITC-CD19 (557398 or 553785, BD Biosciences), APC-CD19 (17-0193-82, eBioscience), PE-CD5 (553022, BD Biosciences), FITC-CD5 (11-0051-82, eBioscience), allophycocyanin-CD1d (17-0011-82, eBioscience), PE-CD1d (12-0011-82, eBioscience), unlabeled rat anti-mouse CD45 (MAB114, R&D Systems) and anti-rat Ig PerCP5.5 (F0115, R&D Systems) and Goat PerCP-Rat F(ab)2 IgG (H+L) (F-0115), for 30 min on ice. For intracellular staining, cells were washed, fixed and permeabilized with Cytofix/Cytoperm solution (554722, BD Biosciences) for 20 min in ice, washed (554723, BD Biosciences) and stained with PerCPCy5.5-FOXP3 (45-5773-82, eBioscience). Cells were washed three times in RPMI medium and re-suspended in 1% formaldehyde for the cytofluorometric analysis. Negative-controls were used to set the flow cytometer photomultiplier tube voltages, and single-color positive controls were used to adjust instrument compensation settings. Cells were examined for viability by flow cytometry using side/forward scatter characteristics or 7-aminoactinomycin D (7-AAD, SML1633, Merck) exclusion. Data (100,000 events acquired per sample) were acquired using a four-color FACSCalibur flow cytometer equipped with CellQuest software (Becton-Dickinson, San Jose, CA). Data were recorded as percent of fluorescent positive cells per organ..

### Assessment of Histological EAE

To evaluate the histological manifestations of EAE, healthy, vehicle-treated EAE, and EAE treated-mice (*n* = 5/group) were killed on days 17 or 30. The spinal cords were removed and fixed in buffered formalin 4%. Paraffin-embedded sections of spinal cord were stained with hematoxylin and eosin (H&E) or with Luxol fast blue (LFB) for analysis of inflammation or demyelination, respectively. Histopathological examination was performed in a blinded fashion.

### Fluoro-Jade C Staining

The 10 micron-thick slices of spinal cord at 17 or 30 day were first deparaffinized in a slice warmer, and then rehydrated through a graduated alcohol series followed by a wash in 70% ethanol. The tissue was briefly rinsed with distilled water and incubated in 0.06% KMnO_4_ solution for 10 min, afterwards, the slices were washed and transferred to a 0.0001% solution of Fluoro-Jade C (AG325, Millipore) dissolved in 0.1% acetic acid for 10 min in dark. The slices were rinsed through three changes of distilled water for 1 min per change. Hundred microliters of 4′,6-diamidine-2′-phenylindole dihydrochloride (DAPI, sc-3000415) solution at 1.5 µg/mL for 10 min were used to label nuclear DNA. The slices were rinsed through three changes of distilled water for 1 min per change. Excess water was drained onto a paper towel, and the slides were air dried on a slide warmer at 37°C for 35 min. The air-dried slices were cover-slipped with 20 µL of Fluoromount G non-fluorescent (00-4958, eBioscience) mounting media and blocked with nail polish. All images were acquired using the OLYMPUS BX51 microscope at a 10x magnification and scale indicates 50 micrometers. The blue nuclear label conferred by DAPI was visualized *via* ultraviolet light excitation, while the Green Fluoro-Jade C staining was quantified by the use of Image J Software v.1.8.0_172. The corrected total cell fluorescence (CTCF) was obtained utilizing the formula CTCF = Integrated Density – (Area of selected cell X Mean fluorescence of background readings).

### Statistical Analysis

All values were expressed as mean ± SEM (standard error of the mean). Experiments using 5 mice per group were performed independently two times. Parametric data were evaluated using analysis of variance, followed by the Bonferroni test for multiple comparisons. Non-parametric data were assessed using the Mann-Whitney test. Differences were considered statistically significant at *p* < 0.05 using GraphPad Prism (Graph Pad Software, v6.02, 2013, La Jolla, CA, USA).

## Data Availability Statement

The original contributions presented in the study are included in the article/**Supplementary Material**. Further inquiries can be directed to the corresponding author.

## Ethics Statement

The animal study was reviewed and approved by Committee on the Ethics of Animal Experiments of the Butantan Institute (#747/10; #1203/14; #1205/14).

## Author Contributions

JB, VZ, EK, and AM contributed to the study design, data collection, analysis, interpretation, and writing. CL and ML-F conceived and supervised the project and writing. All authors contributed to the article and approved the submitted version.

## Funding

This work was supported by the São Paulo Research Foundation -FAPESP [#2012/50.001-0; #2013/07467-1] and in part by the Coordenação de Aperfeiçoamento de Pessoal de Nível Superior – Brasil [CAPES] – Finance Code 001.The funders have no role in study design, data collection and analysis, decision to publish, or preparation of the manuscript.

## Conflict of Interest

The authors declare that the research was conducted in the absence of any commercial or financial relationships that could be construed as a potential conflict of interest.

## Publisher’s Note

All claims expressed in this article are solely those of the authors and do not necessarily represent those of their affiliated organizations, or those of the publisher, the editors and the reviewers. Any product that may be evaluated in this article, or claim that may be made by its manufacturer, is not guaranteed or endorsed by the publisher.
