# Supplementary figures and images for: TnP Peptide Suppresses Experimental Autoimmune Encephalomyelitis (EAE) in a Preclinical Mouse Model
Source: Front Immunol. 2022 Mar 24;13:857692. doi: 10.3389/fimmu.2022.857692 (PMC8988151; doi:10.3389/fimmu.2022.857692)

A)

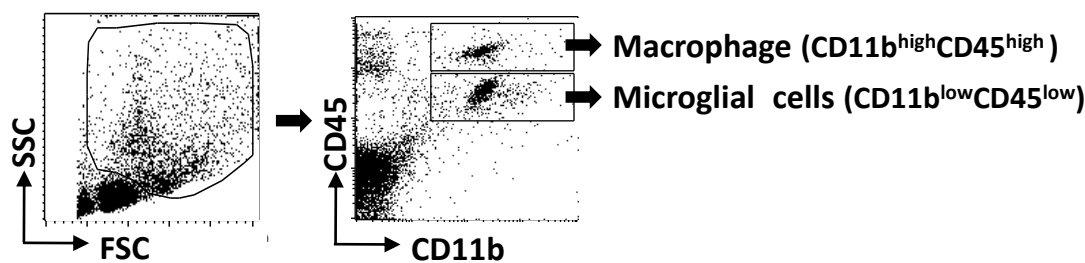

B)

Treg (CD4+CD25+FOXP3+)

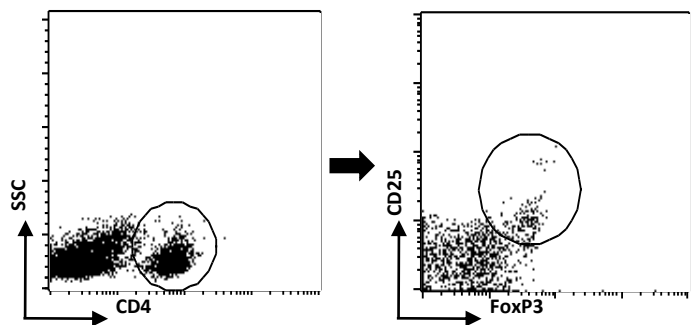

C)

Breg (CD19+CD5+CD1d+)

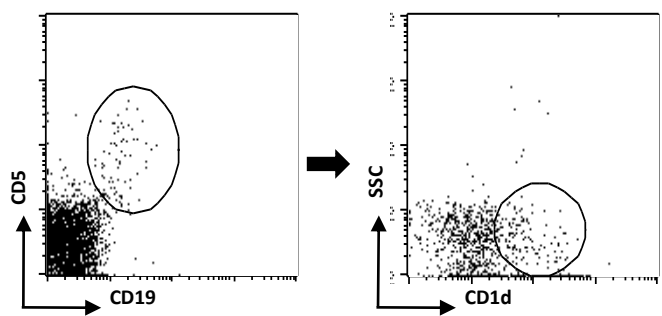

Supplement: Supplementary file 1 [file Image_1.pdf]
